# Gossypiboma Presenting as a Cystic Abdominal Mass: A Case Report

**DOI:** 10.7759/cureus.104740

**Published:** 2026-03-05

**Authors:** Saba Rupani, Amaresh Mishra, Subrat Sahu, Ishwari Suryawanshi, Vedaant Parekh

**Affiliations:** 1 General Surgery, Kalinga Institute of Medical Sciences, Bhubaneswar, IND

**Keywords:** abdominal mass, gossypiboma, medicolegal, retained surgical item, surgical error

## Abstract

Gossypiboma, also known as a retained surgical sponge, is a rare but serious iatrogenic complication following surgery. It often mimics tumors or abscesses and may present months to years postoperatively. This case emphasizes the diagnostic dilemma, medicolegal implications, and the need for prevention. We report a case of a 27-year-old female presenting with a nine-month history of lower abdominal pain and swelling. She had undergone a lower-segment cesarean section (LSCS) 10 months earlier. Imaging revealed a large cystic lesion initially suspected to be a hydatid cyst. Exploratory laparotomy uncovered a gossypiboma: a retained surgical mop encased in a cystic mass. The mass was excised, and the patient recovered uneventfully. Gossypiboma should be considered in the differential diagnosis of unexplained postoperative masses. Prevention through meticulous intraoperative protocols remains the best approach, as the consequences are serious and avoidable.

## Introduction

Gossypiboma, also referred to as textiloma or retained surgical sponge, is an iatrogenic condition resulting from inadvertent retention of surgical materials, most commonly cotton sponges or mops, within the patient’s body after an operative procedure. The term is derived from the Latin *gossypium* (cotton) and the Kiswahili* boma* (place of concealment). Although considered rare, gossypiboma represents a serious breach in surgical safety and is classified as a “never event,” as it is theoretically preventable through adherence to standardized intraoperative protocols [1,2]. The true incidence is likely underestimated due to underreporting related to medicolegal concerns, with reported rates ranging from one in 1,000 to one in 5,000 abdominal surgeries [3-5]. From a clinical standpoint, gossypiboma exhibits a wide spectrum of presentations, largely influenced by the host inflammatory response and the duration of retention. Two principal pathological reactions have been described. The acute exudative response is characterized by intense inflammation, leading to abscess formation, localized peritonitis, sepsis, or fistula formation, typically manifesting in the early postoperative period [3,6]. By contrast, the chronic aseptic fibrotic response involves gradual encapsulation of the retained sponge by granulation and fibrous tissue, resulting in a mass lesion that may remain asymptomatic for months or even years [3,7]. Patients may present with nonspecific symptoms such as abdominal pain, distension, fever, weight loss, or a palpable mass, making clinical diagnosis particularly challenging. Gossypiboma is notorious for mimicking a variety of intra-abdominal pathologies, earning its reputation as a “great mimicker.” Differential diagnoses include neoplastic masses, parasitic cysts (notably hydatid disease in endemic regions), chronic abscesses, hematomas, and postoperative adhesions [8-10]. Such overlap in clinical and radiological features often leads to extensive diagnostic workups and delayed definitive management.

Awareness of prior surgical history is therefore critical, especially in patients presenting with unexplained masses following abdominal or pelvic surgery. Radiological investigations play a pivotal role in the evaluation of suspected gossypiboma, although findings may be nonspecific. Ultrasonography may demonstrate a cystic or hypoechoic mass with internal echogenic wavy or striped structures. Computed tomography (CT) is generally considered the imaging modality of choice and may reveal a well-defined mass with a spongiform appearance, internal whorled densities, mottled gas patterns, or a thick fibrous capsule [11-13]. Magnetic resonance imaging (MRI) findings are variable and depend on the degree of fibrosis, fluid content, and inflammatory reaction, at times leading to misinterpretation as malignant or parasitic lesions [14]. The absence or degradation of radiopaque markers further compounds diagnostic difficulty. Management of gossypiboma is primarily surgical, with complete removal of the retained material and surrounding inflammatory tissue being the treatment of choice. Delayed diagnosis can result in serious complications, including bowel obstruction, perforation, fistula formation, sepsis, and, in rare cases, mortality [7,15]. Given the significant clinical, ethical, and medicolegal implications, prevention remains paramount. Identified risk factors include emergency procedures, unexpected intraoperative events, prolonged surgeries, obesity, and inaccurate sponge counts, factors commonly encountered in obstetric and gynecological surgeries, such as cesarean sections [1,2,6]. Modern preventive strategies emphasize a systems-based approach, incorporating standardized counting protocols, radiopaque sponges, adjunct technologies such as barcode or radiofrequency identification systems, and structured safety tools, like the World Health Organization Surgical Safety Checklist [16-18].

## Case presentation

Patient information

A 27-year-old female presented with complaints of abdominal swelling and pain for nine months. She had no prior history of chronic illness. Her surgical history included a lower-segment cesarean section (LSCS) performed in May 2023.

Clinical findings

**Figure 1:**
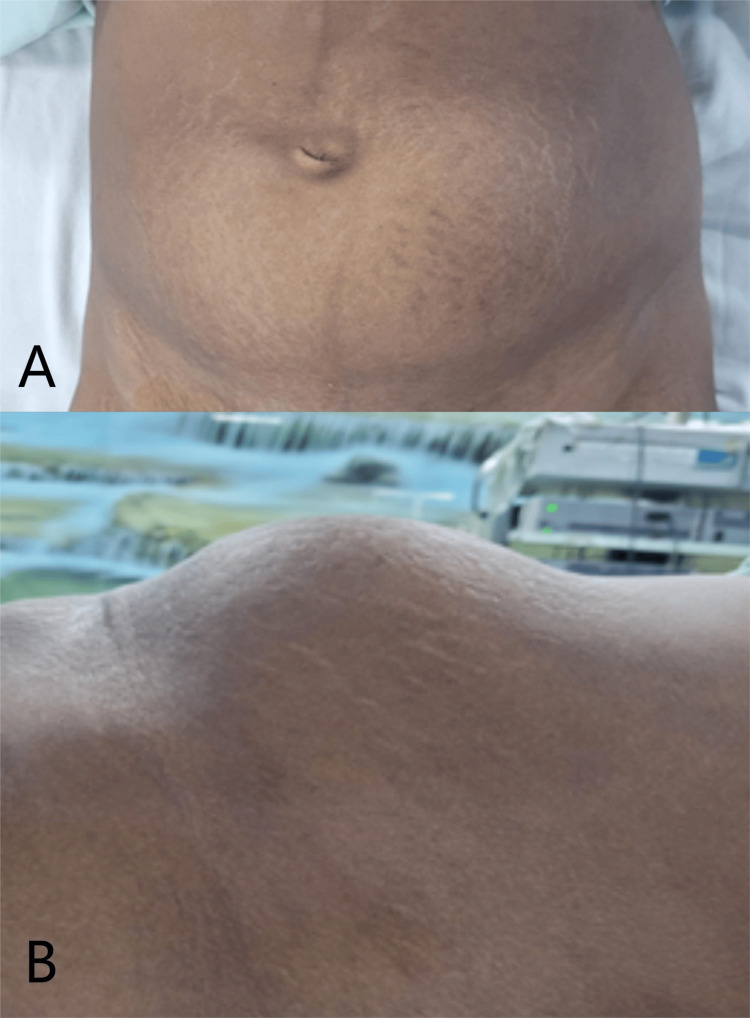
Abdominal swelling (A) Anterior abdominal view demonstrating significant lower abdominal swelling. (B) Lateral view highlighting the extent of abdominal distension.

On examination, her abdomen was distended. The umbilicus was displaced to the right and inverted. A healed LSCS scar was visible. Palpation revealed a firm, smooth, well-defined mass measuring approximately 10 × 15 cm in the suprapubic region, as seen in Figure 1. Mild tenderness was noted over the swelling.

Timeline

The timeline of events is described in Table 1.

**Table 1:** Timeline of events LSCS: lower-segment cesarean section, CECT: contrast-enhanced computer tomography

Date	Event
05/2023	LSCS performed
12/2023	Onset of abdominal pain and swelling
04/2024	Ultrasound abdomen/pelvis suggests hydatid cyst
04/2024	CECT showed laminated cysts ("water lily sign")
04/2024	Exploratory laparotomy performed, gossypiboma found

Diagnostic assessment

**Figure 2:**
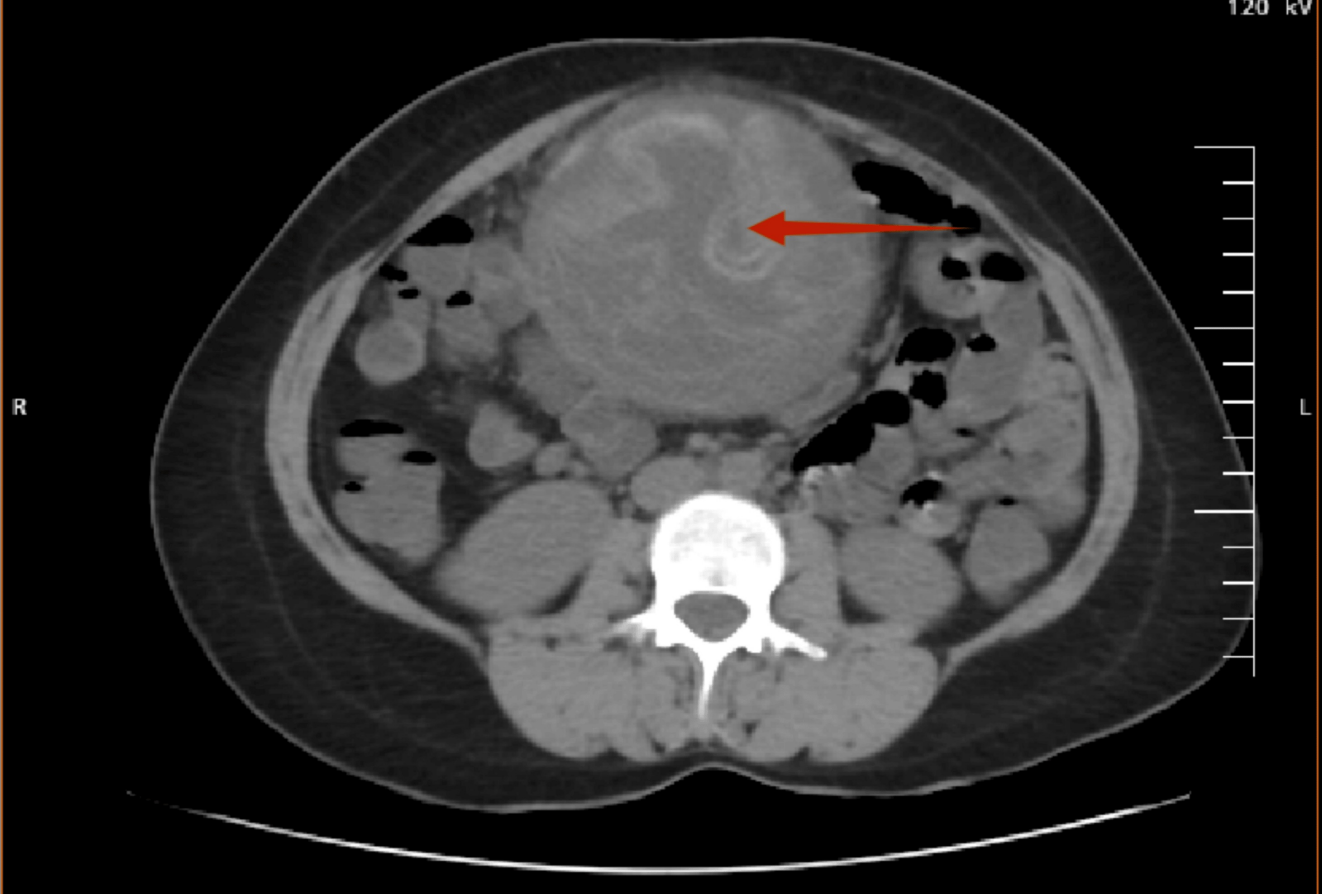
"Water lily" sign on CECT, marked with an arrow CECT: contrast-enhanced computer tomography

Contrast-enhanced computer tomography (CECT) abdomen (done in April 2024) suggested daughter cysts and laminated membranes, showing the characteristic "water lily" sign, consistent with a hydatid cyst, as seen in Figure 2. *Echinococcus* IgG was negative (2.04 IU/ml), indicating a less likely chance of echinococcal infection.

Therapeutic intervention

**Figure 3:**
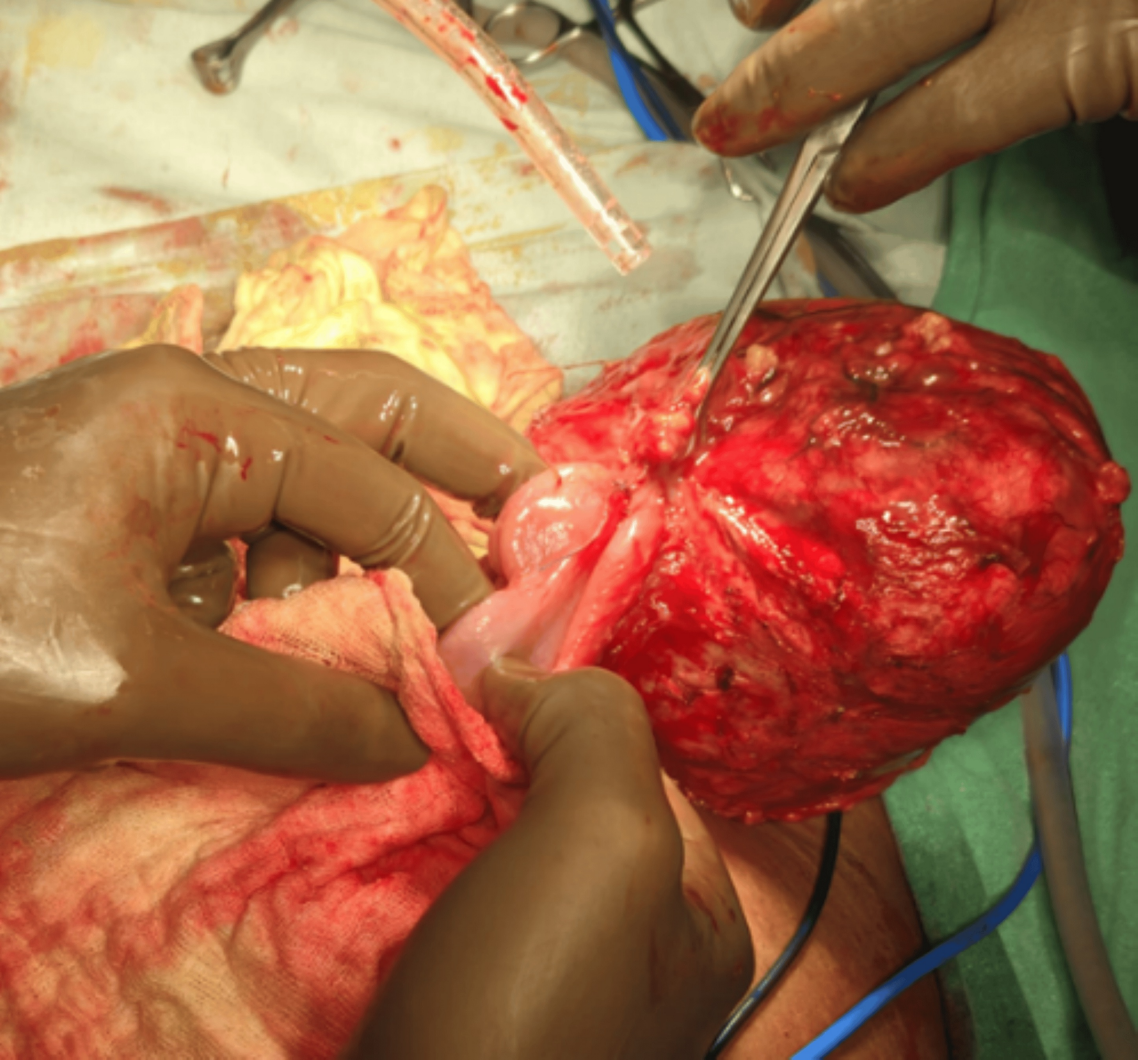
Cyst as seen intraoperatively

**Figure 4:**
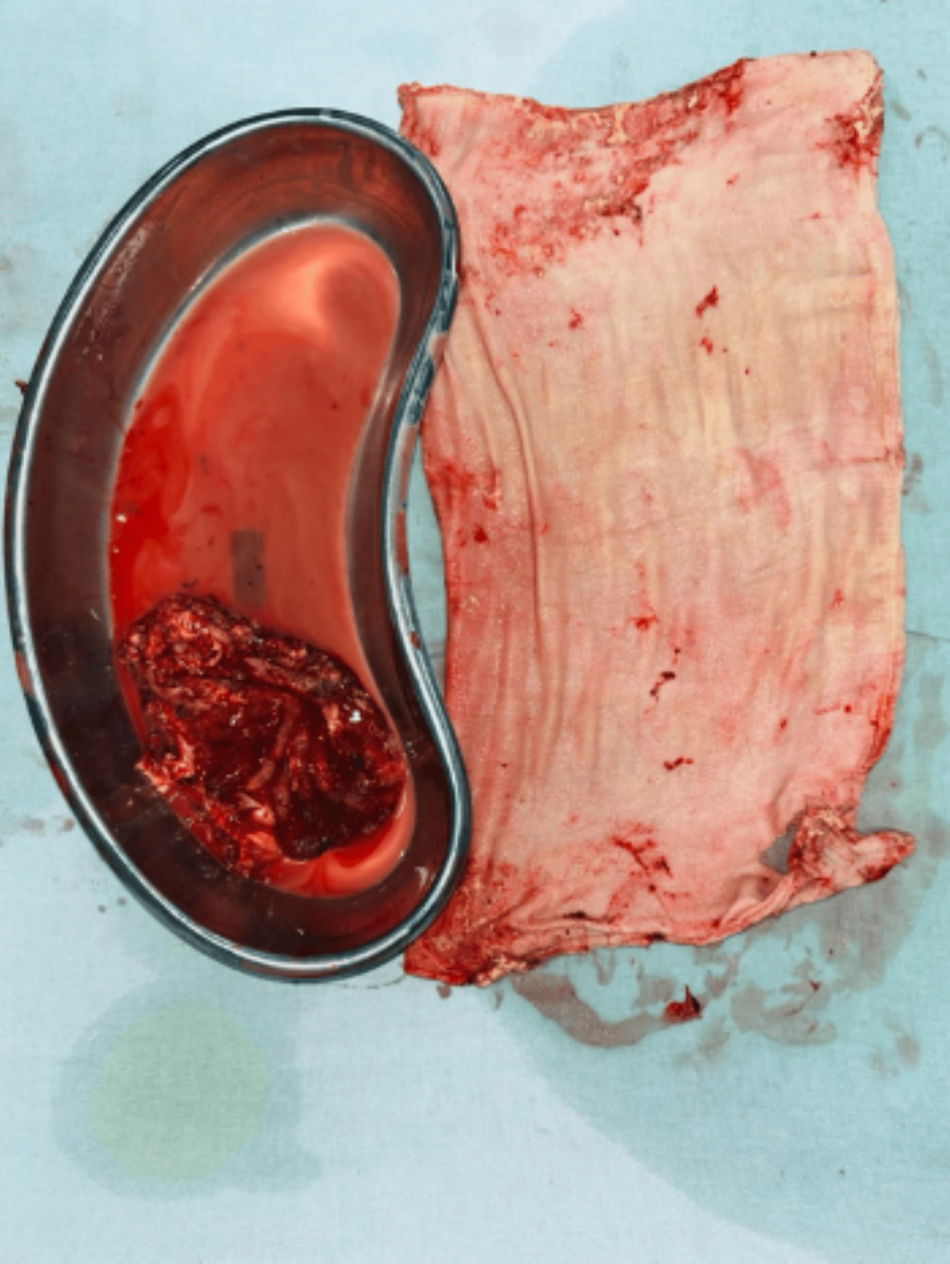
: Excised cyst wall and retained surgical mop specimen

An exploratory laparotomy was performed. Intraoperatively, a 10 × 15 cm cyst was found in the lower abdomen (as seen in Figure 3), adherent to the small bowel wall without any infilteration intramurally approximately 50-70 cm proximal to the ileocecal junction. On incision, the cyst contained thick pus and a retained surgical mop. The mass was excised completely (as seen in Figure 4) and sent for histopathological evaluation.

**Figure 5:**
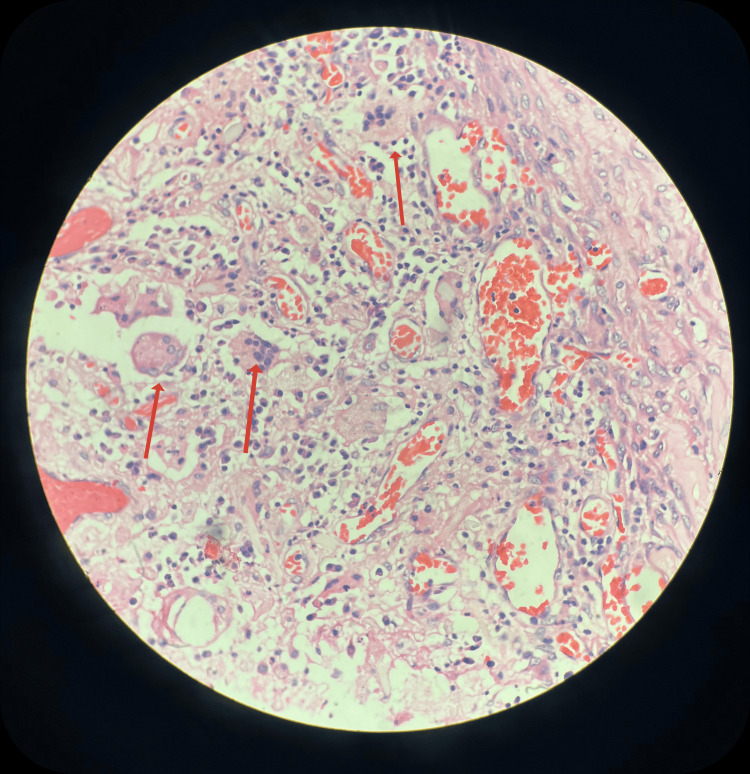
Histopathological features of the specimen Photomicrograph showing cyst wall with dense chronic inflammatory infiltrate, sheets of foamy histiocytes, and multinucleated foreign body-type giant cells (arrows). (H&E stain, original magnification ×400).

Histopathological evaluation revealed a cyst wall lined by a band of inflammatory cells, foamy histiocytes, and a foreign body engulfed by giant cells, as seen in Figure 5. This confirmed the diagnosis of gossypiboma.

Follow-up and outcomes

The cyst was densely adherent to the adjacent bowel loops, and meticulous dissection was performed to prevent injury to surrounding structures. The cyst, along with the retained surgical sponge (gossypiboma), was completely excised in toto. There was no intraoperative bowel injury or damage to adjacent organs, and hemostasis was adequately secured.

The postoperative course was uneventful. The patient recovered well without any immediate or delayed complications and was discharged on postoperative day 5. Follow-up evaluations at two weeks and six weeks, and subsequently at six months, were unremarkable, with complete resolution of symptoms and no evidence of recurrence or residual intra-abdominal pathology.

## Discussion

Gossypiboma, also known as textiloma or retained surgical sponge, is a rare but serious iatrogenic complication that continues to challenge modern surgical practice despite advances in operative techniques and patient safety protocols. Retained surgical items (RSIs) are considered “never events,” as they are theoretically preventable through standardized intraoperative safeguards. Nevertheless, sponges remain the most commonly retained foreign bodies, accounting for the majority of reported RSI cases across surgical specialties [1,2]. The actual incidence is likely underestimated due to fear of litigation and reputational harm, with reported rates ranging between one in 1,000 and one in 5,000 abdominal operations [3].

The biological response to a retained sponge is influenced by the host immune reaction and the duration of retention. Two distinct pathological patterns have been described. The acute exudative type is characterized by intense inflammatory reaction leading to abscess formation, localized peritonitis, fistulization into adjacent organs, or sepsis, often resulting in early postoperative symptoms [3,6]. In contrast, the chronic aseptic fibrotic type involves gradual encapsulation of the sponge by fibrous tissue, forming a mass that may remain clinically silent for months or even decades [3]. The present case exemplifies this latter response, with the retained mop evolving into a cystic encapsulated lesion presenting nine months after cesarean delivery.

Clinically, gossypiboma has earned a reputation as a “great mimicker.” Patients may present with nonspecific symptoms such as abdominal pain, distension, fever, or a palpable mass, depending on the site and chronicity of the lesion. Numerous reports describe gossypibomas masquerading as malignant tumors, parasitic cysts, or chronic abscesses, often leading to extensive diagnostic workups and delayed definitive management [8]. In the current case, imaging features suggestive of a hydatid cyst, including laminated membranes and cystic architecture, demonstrate how convincingly gossypiboma can mimic parasitic disease, particularly in endemic regions.

Radiological evaluation plays a central role in the diagnostic process, yet remains fraught with pitfalls. Ultrasonography may reveal hypoechoic or cystic lesions with internal echogenic wavy structures, while computed tomography is considered the imaging modality of choice. Typical CT findings include a well-defined mass with a spongiform appearance, mottled gas patterns, internal whorled densities, or a thick fibrous capsule [11]. However, these classic features may be absent, especially when radiopaque markers are missing or have degraded over time, rendering diagnosis difficult [14]. Magnetic resonance imaging further adds variability, as signal characteristics depend on fluid content, fibrosis, and inflammatory changes, occasionally leading to misinterpretation as neoplastic pathology [14].

Risk factors for retained surgical sponges have been extensively studied. Emergency operations, unexpected changes in surgical plans, prolonged procedures, obesity, inadequate communication, and inaccurate sponge counts consistently emerge as significant contributors [2,6]. Obstetric surgeries, particularly cesarean sections performed under urgent or emergent conditions, are among the most frequently implicated procedures, making the present case clinically and epidemiologically relevant [1,2]. These findings reinforce the concept that RSIs are rarely the result of isolated individual error, but rather represent complex system-level failures.

The medicolegal and ethical dimensions of gossypiboma are substantial. Retained surgical sponges are commonly litigated under the doctrine of *res ipsa loquitur*, as their presence implies negligence without the need for further proof [13]. Such cases can result in significant legal penalties, loss of professional credibility, and erosion of patient trust. Importantly, modern patient safety literature advocates shifting the focus from individual blame to systemic accountability, emphasizing the redesign of processes and operating room culture to prevent recurrence [6,16].

Prevention remains the most effective strategy for addressing gossypiboma. Traditional measures such as manual sponge counts and radiopaque markers, while essential, are vulnerable to human error. As a result, adjunct technologies, including barcode-based sponge tracking systems and radiofrequency identification (RFID) devices, have been developed and shown to significantly reduce the incidence of retained sponges [16,17]. Furthermore, structured safety interventions such as the World Health Organization Surgical Safety Checklist and national surgical quality improvement initiatives have demonstrated measurable improvements in communication, teamwork, and compliance with safety standards [18]. These system-wide approaches underscore that elimination of RSIs is an achievable goal when prevention is embedded into the surgical workflow.

This case adds to the growing body of literature highlighting the diagnostic complexity and potential morbidity associated with gossypiboma. It reinforces the necessity of maintaining a high index of suspicion in postoperative patients presenting with unexplained abdominal masses and underscores the enduring importance of vigilance, accountability, and system-based preventive strategies in surgical practice.

## Conclusions

Gossypiboma is a rare yet entirely preventable postoperative complication with significant clinical, psychological, and medicolegal implications. This case highlights the diagnostic challenges posed by retained surgical sponges, particularly when imaging findings mimic other pathologies such as hydatid cysts or neoplasms, as seen in this patient who presented months after cesarean section with an abdominal mass initially suggestive of a hydatid cyst. Such diagnostic ambiguity underscores the importance of maintaining a high index of suspicion in patients with a prior surgical history, even when radiological features favor more common regional differentials.

Definitive diagnosis is frequently established intraoperatively and confirmed histopathologically, with complete surgical excision remaining the cornerstone of management. Early recognition and timely intervention are crucial to prevent complications, including infection, fistula formation, bowel obstruction, and sepsis.

However, the true solution to gossypiboma lies in prevention rather than treatment. Strict adherence to standardized sponge and instrument counts, effective intraoperative communication, routine use of radiopaque materials, implementation of surgical safety checklists, and adoption of adjunct detection technologies are essential measures to minimize risk. RSIs should be recognized as system failures rather than isolated individual errors, necessitating a culture of safety, accountability, and transparency within healthcare institutions.

By reporting this case, we aim to raise awareness among surgeons, radiologists, and clinicians regarding the varied presentations of gossypiboma and to reinforce the ethical and professional responsibility to implement robust preventive strategies. The elimination of RSIs is both achievable and fundamental to ensuring surgical quality and patient safety.
